# A framework for improved one health governance and policy making for antimicrobial use

**DOI:** 10.1136/bmjgh-2019-001807

**Published:** 2019-09-18

**Authors:** Dominic Moran

**Affiliations:** Global Academy of Agriculture and Food Security, The Royal (Dick) School of Veterinary Studies and The Roslin Institute, The University of Edinburgh, Edinburgh, UK

**Keywords:** antimicrobial use, governance, One Health, economics

## Abstract

There is a need to develop an evaluation framework to identify intervention priorities to reduce antimicrobial use (AMU) across clinical, agricultural and environmental settings. Antimicrobial resistance (AMR) can be conceptualised and therefore potentially managed in the same way as an environmental pollution problem. That is, over-use of antimicrobial medicines as inputs to human and animal health leads to unintended leakage of resistance genes that further combine with natural or intrinsic resistance in the environment. The diffuse nature of this leakage means that the private use decision is typically neither cognisant, nor made responsible for the wider social cost, which is the depletion of wider antibiotic effectiveness, a common pool resource or public good. To address this so-called market failure, some authors have suggested a potential to learn from similar management challenges encountered in the sphere of global climate change, specifically, capping use of medically important drugs analogous to limits set on greenhouse gas emissions. Drawing on experience of the economics of greenhouse gas mitigation, this paper explores a potential framework to develop AMU budgets based on a systematic comparative appraisal of the technical, economic, behavioural and policy feasibility of AMU reduction interventions across the One Health domains. The suggested framework responds to a call for global efforts to develop multi-dimensional metrics and a transparent focus to motivate research and policy, and ultimately to inform national and global AMR governance.

**Summary box**

- There are many interventions to reduce antimicrobial use (AMU), but there is no common framework for their prioritisation on the basis of cost-effectiveness
- Conceptualised as an environmental pollution problem, AMU can be managed using analogous approaches used to reduce global greenhouse gas emissions.
- An abatement cost curve framework offers a systematic approach for data collection across the One Health settings, with a view to setting rational AMU targets
- Policy interventions need to identify and prioritise the most cost-competitive interventions to modify AMU across all settings.
- Global governance of AMR will benefit from a transparent approach to guide data collection for global and national action planning.

## Introduction

The biological bounty of antibiotic discovery is measurable in terms of the avoided human and animal health burden over more than six decades. As noted in countless reports and papers, and indeed as originally foreseen, this period is potentially ending with the prospect of increased multi-drug resistant infections born of poor stewardship and over use of an existing suite of drugs, and supply side barriers inhibiting innovation of replacements.^1^

A nexus of human, animal and environmental origin, antimicrobial resistance (AMR), and its driver antimicrobial use (AMU), is a quintessential One Health challenge. The biologically complex properties of AMR are well documented and have been likened to similar wicked diffuse environmental pollution problems, and the challenge of reducing global greenhouse gas (GHG) emissions in particular.^2–4^ The parallels are indeed striking: AMR is characterised by market and other institutional (ie, common property) failures in terms of resistance costs being externalised nationally and globally by injudicious private use. There are multiple human and animal sources of resistance ‘pollution’, further complicated by complex interactions, pooling and persistence in the environment. There are multiple potential entry points to modify clinical and veterinary uses of medicines, to intercept environmental pollution, and for detection and diagnosis of resistance. Some interventions can complement or interact in unanticipated ways. There is an obvious human dimension to AMU, raising unresolved behavioural challenges in the face of regulatory alternatives. Finally, there are obvious mitigation (or abatement) cost and political economy dimensions, pitting public and private sector interests, and dividing governance objectives in upper and lower and middle-income countries. One key difference however is that there is currently no global externality cost metric for AMR similar to a carbon price to facilitate cost-benefit analysis for GHG emissions mitigation. However, this is not a barrier to cost-effectiveness analysis, which is the focus of this paper.

As a regulatory response, global AMR action plans offer only the broadest parameters around the notion of good antimicrobial stewardship.^5 6^ They do not yet set a clear pathway for appraising mitigation actions, and arguably for defining efficient policy priorities. Recent scrutiny of governance structures^7 8^ has suggested converting the UN tripartite (WHO, World Animal Health Organisation, Food and Agriculture Organisation of the United Nations) into a standing secretariat of a new Global Multi Stakeholder Steering Board that would direct a more coherent science policy programme. This suggestion echoes an earlier call to develop an intergovernmental panel on antimicrobial resistance, based on the to the Intergovernmental Panel on Climate Change (IPCC) process, which has been instrumental in developing cost-effective interventions on GHG mitigation, adaptation and equitable north–south pathways for decarbonisation.

Links between AMU and AMR are complex, but there is growing consensus that AMU is a driver of resistance pressure, and that the common property and market failures need to be a focus of attention alongside drug innovation. As noted by Hoffman *et al*,^9^ innovation without conservation will waste new drugs and diminish the value of investments. That is, there needs to be a simultaneous focus on both demand and supply sides of the problem.

The climate change experience can be instructive and there is value in exploring how the analogy of a GHG emissions cap may translate into the context of managing AMU. Drawing on experience in developing UK GHG budgets,^10 11^ we suggest that any new scientific collaboration to improve governance must be based on a consistent and systematic framework to organise data across all uses, and to consider a range of economic, behavioural and policy barriers that will define the suite of feasible interventions on AMU. We propose a marginal abatement cost curve (MACC) as a shared framework and a focus for any new scientific collaboration to develop evidence-based targets and multi-dimensional metrics to identify a clearer way forward.^7^ This suggestion responds to a call^8^ summarising the work of the ad hoc Interagency Coordination Group (IACG) on global AMR governance; among other recommendations, suggesting a cap on use and requesting suggestions for roles for a putative Governance Board. The paper is organised as follows. The Governance challenges section reflects further on governance challenges, the parallels between climate change mitigation and AMR, and lessons from the application of marginal abatement cost theory, which derived from the economics of pollution control. The Marginal abatement cost theory and AMU section briefly outlines criteria that arise in constructing a MACC, the Applying the marginal abatement cost curve framework section provides a discussion and conclusion.

## Governance challenges

The alarm around rising drug resistant infection and scrutiny of governance structures has highlighted overlapping roles and gaps in existing multi-lateral structures and national strategies, and the need to seek consensus on both the magnitude of the problem and national responses.^7 12 13^ Emphasis on legally binding mechanisms^14^ is a necessary but insufficient condition to regulate use within global and national limits (ie, caps). However, policies and caps needs to be underpinned by a transparent framework that can accommodate several criteria relevant to the limitation on drug use, not least the full economic costs of implementing interventions in different contexts.

Different metrics and frameworks have been advanced to communicate and help regulate complex AMU/AMR links within and across One Health settings. These include systems mapping and models,^15 16^ The Driver-Pressure-State-Impact-Response framework, Socio-Ecological System and resilience thinking,^12 17^ an Ecosystem Approach,^18^ safe operating spaces and planetary boundaries,^19^ integrated One Health surveillance systems^20^ and antibiotic footprinting.^21^ Most draw on experience in managing environmental pollution, but none leads to a clear picture on priority setting for mitigation or the basis for a cap based on the relative cost of interventions. In other words, if we are targeting AMU in a specific setting (eg, agriculture), and within a specific jurisdiction, what criteria and by extension what data, might we use to identify priority measures and the extent of their implementation?

This situation is reminiscent of the climate change experience. A decade ago, there was much unstructured discussion and data uncertainty around different GHG mitigation measures, but a limited sense of the lowest cost ways to avoid carbon dioxide equivalent emissions across different sectors. In seeking to identify their contribution to global emissions reduction targets, some governments took the initiative to develop a more rational approach to sectoral carbon budgeting. These ranked cost competitive mitigation interventions cheapest to most expensive (per unit reduction in emissions), in each sector, thereby facilitating comparison of abatement costs across sectors. A comparison of these costs, across all emitting sectors reveals where the next (marginal) abatement intervention should notionally be implemented. If some upper marginal abatement cost limit is defined, then this process illustrates the overall extent of emissions mitigation that is economically feasible for each sector and in aggregate. With this information, it is possible to identify the relative contribution of different sectors and implicitly the remaining emissions envelope within each, a carbon budget, which would be excessively expensive to reduce. Cost in this context means the private cost of compliance associated with the intervention, but can also include wider assessment of life cycle and social (ie, environmental) costs and benefits that can be incurred when inserting a technology into a production system.

## Marginal abatement cost theory and AMU

The abatement cost logic derives from basic economic theory of pollution control, which for point source pollution models suggests that an allocation of emissions among polluters is efficient if it minimises the costs of achieving an ambient environmental target.^22^ Any IPCC-like process applied to AMU caps will eventually need to address the logic of economic efficiency as a starting point for developing AMU budgets.

Conceptualised as diffuse pollutants, AMR and GHGs share similar properties that can be explored using marginal abatement cost theory of optimal pollution abatement. Given the complex nature of AMR in the environment, we are more pragmatically applying this to AMU as a precursor or potential interventions that reduce the pressure of environmental pollution. We apparently know little about the relative cost-effectiveness of mitigating AMU or reducing environmental exposures across One Health settings; that is, in clinical, agricultural, and environmental spheres. Learning from climate change, this is arguably the most conspicuous gap for economists to help bridge the disciplinary divides and reframe the challenge in a way that provides a clear way forward for policy and governance.

**Figure 1:**
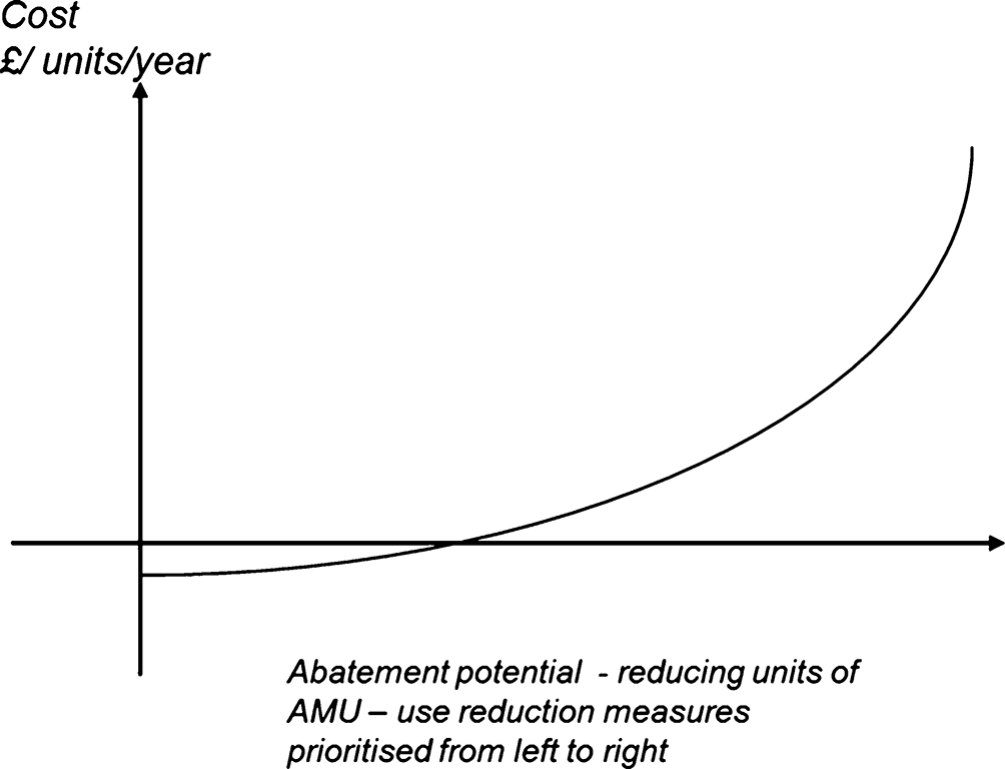
Theoretical Marginal Abatement Cost Curve. AMU, antimicrobial use.

If society wants to prioritise lowest cost AMU reductions, for each sector there is a theoretical schedule that traces out the increasing marginal cost of units of pollution reduction (figure 1). At the upper end, extra pollution abatement is expensive. In contrast, the lower end, below the x axis, implies that in some sectors there is a theoretical cost saving in reducing pollution. This can come about when a polluting input is actually being over-used. This might be the case in terms of livestock diseases prophylaxis or growth promotion, or else in some clinical contexts where antibiotics are inappropriately prescribed for misdiagnosed conditions. There are some interesting behavioural explanations for why such cost-saving measures might not be automatically recognised and adopted, which will be returned to presently.

**Figure 2:**
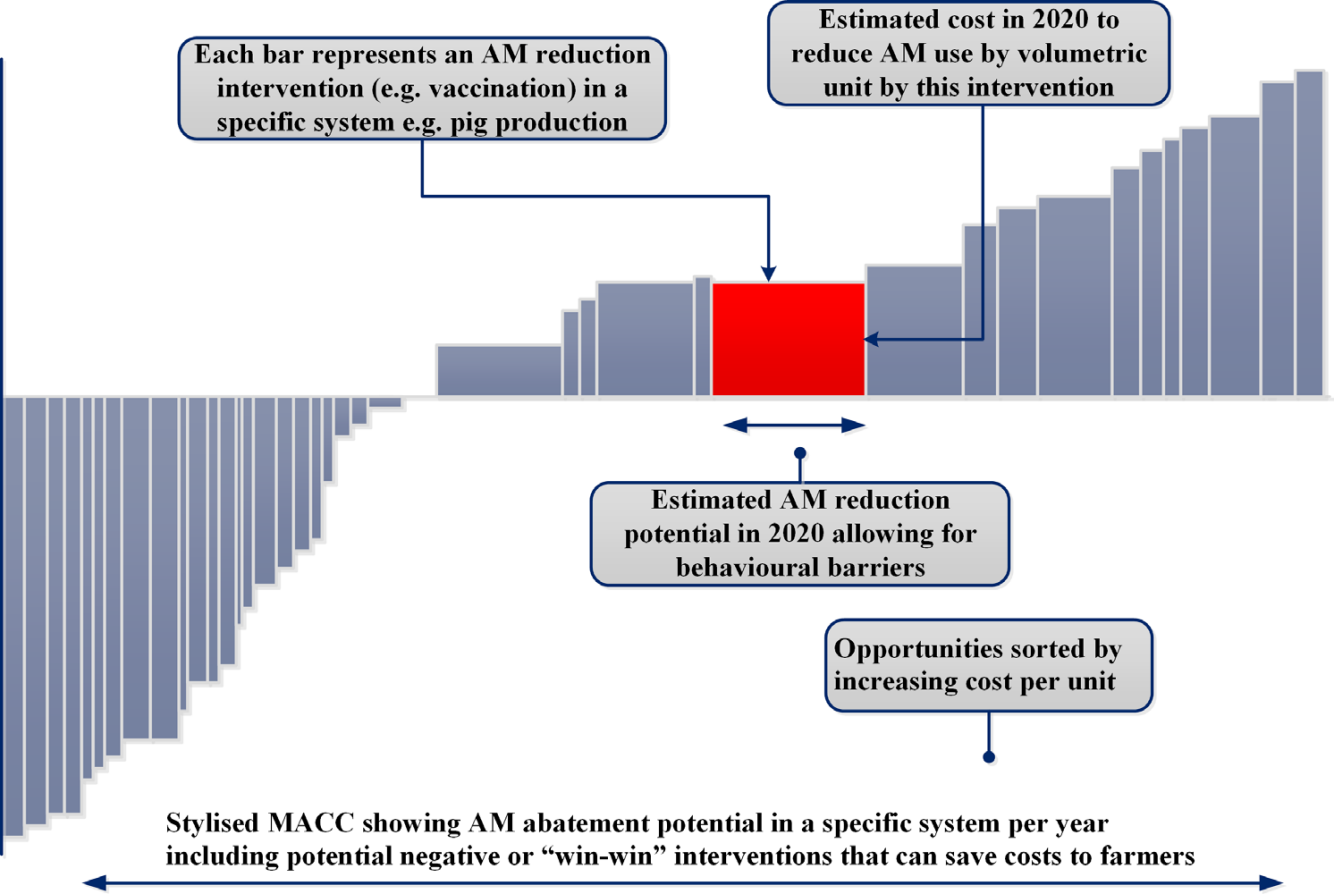
Bottom up abatement cost schedule. AM, antimicrobial; MACC, marginal abatement cost curve.

Translating this theory into practice requires a bottom up variant of the MACC approach that recognises that the schedule is not comprised of infinitely divisible homogeneous interventions implied by the curve in figure 1. Instead, each intervention represents a known technology, system change, or behavioural intervention to affect AMU, applicable to a production or drug administration process. In a specific sector or working across sectors, translation implies a series of analytical steps that ultimately represent each mitigation measure as a bar (figure 2) indicating its mitigation potential (width)—the amount of AMU reduction afforded by its implementation, and the implicit mitigation cost (per relevant unit) per year.

Estimating this information within a sector implies a series of analytical steps, approximately summarised to:

- identify all known technical mitigation interventions or measures that could apply in a setting;
- understand how each works and their current (baseline or business as usual) use or research potential;
- the extent to which each measure can be applied alone or combined, and with what approximate forecast outcome in terms or reduced AMU or reduction in emissions to the environment relative baseline trajectory;
- calculate financial implementation costs plus wider economic and ancillary costs and benefits derived from implementation;
- identify which measures are relatively cost-effective including those that are win-win; that is, implying negative or cost savings;
- identify which available policy instruments apply to which measures;
- identify measures that need further research, are relatively uncertain, currently out of scope due to unproven technical effectiveness, excessive cost or policy/regulatory feasibility;
- identify measures with behavioural barriers that can reduce technical, economic and policy feasibility.

MACCs are typically derived at scales meaningful in terms of governance and policy jurisdiction, and some of the more galvanising messages come from analysis at the global scale.^23^ Note that while we are initially considering demand side measures (ie, AMU in the production process), there is no reason to exclude integrated supply chain and consumption side measures that can be affected by policy. For example, awareness and advertising around certain animal (food) products or many healthcare interventions that seek to affect behavioural change in prescribing or drug consumption. Quantifying the cost-effectiveness of these measures is challenging, but in clinical and agricultural spheres, there is evidence of their effectiveness.^13 24^

Stages (i)–(viii) frame and summarise an interdisciplinary science-policy research agenda that can provide different potential abatement estimates as policy targets. A full technical potential is the mitigation opportunity available in a sector where all technically feasible measures are fully applied over a given time horizon. This potential is a largely theoretical benchmark due to applicability constraints on some technologies, which may rule them out on technical, economic or ethical grounds. In practice, the focus is on the economic potential, which comprises the total abatement of the lowest cost or most cost-effective measures. As noted, there is no analogous pollution price (ie, a carbon price) benchmark to set a cut-off point for excessive cost,^4^ but in specific settings, this decision can be informed by consensus or rulings around the concept of Best Available Technology Not Entailing Excessive Cost.

The economic potential is in turn limited by the fact that there may be insufficient policy levers to implement (incentivise or mandate) application of some measures. Again, the framework serves to focus attention on policy design. This remaining ‘policy-feasible’ potential is finally limited by potential behavioural barriers that mean that measures are not adopted as anticipated.

These potentials can be increased by measure-specific research spanning the science-policy continuum. As noted previously, in relation to potential cost-saving interventions, there are demonstrable behavioural anomalies that impede the adoption of even cost saving measures. In this case, insights can be derived from both psychology and behavioural economics.^25^ Ultimately, once the feasible abatement potential is identified as a target, the allowable budget is identified by deducting the achievable abatement potential from the projected baseline trajectory derived in stage (ii). The budget is then a targeted for year on year monitoring and progress.

## Applying the MACC framework

Applying a MACC framework implies a consistent multi-disciplinary research agenda to define cost-effective interventions across the One Health space. In the climate change context, MACCs have focused debate around a common problem framing and methodology that has galvanised science to identify gaps and refine assumptions relevant to each measure, and to understand immediate versus less proximate priorities for accelerating cost-effective abatement potential. This includes research on integrated modelling, behavioural barriers and a focus on the relative effectiveness of measure-specific policies. Note that there is no suggestion of a definitive MACC; rather that the exercise provides an estimate that is continually improved as new data emerge and are captured. The UK agricultural GHG MACC is in its sixth iteration since 2008 and is in some senses an exemplar of multi-disciplinary science-policy collaboration.

Table 1 outlines exemplar interventions in clinical, agricultural and environmental settings. The list is not intended to be exhaustive and there are likely to be hundreds of technological and behavioural interventions across these settings, from which a short listing is necessary in stage (i) (eg, through participatory Delphi and or multi-criteria methods). Considering the scope for AMU reduction in a specific jurisdiction, and with a view to action planning, a thought experiment is to consider whether it is currently possible to rank any of these measures in terms of the potentials outlined above. In other words, without framing the objectives clearly, there is significant potential for implementing policy inefficiently.

**Table 1:** Measures to modify AMU

Health/clinical	Agriculture and food	Water, sanitation and environment
Adherence to therapies Integration of new diagnostics/therapy strategies Effective health messaging and information Promotion of antibiotics Public health messages Voluntary/mandatory/market-based incentives Appropriate access Issue mainstreaming Experience of treatment campaigns Human microbiome, nutritional status, coinfection Available antibiotics Drug combinations vs monotherapies Drug dosage Frequency of dosage Packaging design Syndromic treatment Prophylactic treatment Universal health coverage Deferred prescriptions Distinctive labelling Patient screening and isolation Watchful waiting	Nudging prescription habits Breeding and precision editing Microbiome manipulation Vaccines/disease eradication Phage and Lysins Immunomodulation Novel antimicrobials In feed enzymes Prebiotics and probiotics Phytochemicals (eg, essential oils) Biosecurity/heard health planning Husbandry Housing and animal welfare Shift away from growth promoters Training veterinary medics in importance of responsible antibiotic use Improved biosecurity at farms Rapid and cheap diagnostics Restrictions on highly critical antibiotics Peptides Antibodies Improve carcass and food handling Reduce effluents from farms	Improved sanitation Improved access to clean water Primary, secondary and tertiary water treatment Regulatory standards on discharges of antibiotics from production facilities including determination of differentiated maximum admissible levels of an antibiotic, ARB or ARG Farm waste management membrane filtration, activated carbon bio filters, photo-driven technologies and ozonation, wetland-treatment Infection prevention and control

AMR is biologically complex and there will be significant heterogeneity in the cost-effectiveness of measures implemented indifferent contexts. Focusing on AMU and interventions that reduce environmental pollution side steps uncertainties in managing AMR,^26^ but there is value in a pragmatic process that builds scientific consensus to prioritise AMU interventions. Indeed, it is hard to develop a meaningful conversation about global governance, stewardship and capping use, until the world develops evidence on notional and equitable targets underpinned by a similar approach. MACCs allow us to frame relevant questions that we should be asking in action planning in upper, middle and lower income countries. In the latter, they can assist targeting for development assistance. That is, they are a basis for a discussion about the equity of which measures can be implemented, where, with and without external assistance. This discussion is akin to the climate change negotiations that distinguished between mandatory targets for so-called Annex 1 (developed) countries and voluntary commitments by Annex 2 (developing countries) pending aid for mitigation and adaptation. The nature of any transformative change in AMU can equally be framed with reference to the rhetoric around just transitions in the context of decarbonisation and development.^27^

More immediately, they respond to several recommendations set out by IACG.^7^ Specifically, A2 on action planning and prioritised actions, B3 a coordinated global mapping of research and development activities, E1 calling on tripartite group to reinforce capacity for target setting, E3 which suggests the generation (by a scientific panel) of evidence on mitigation and adaptation, and finally as part of a global stewardship framework E4.

## Conclusion

Current AMR governance spans organisations sharing no obvious analytical frameworks to cumulate data systematically over the different One Health settings to understand AMU reduction opportunities and targets. Although advocated,^7^ there is as yet no obvious methodological approach to deriving an AMU cap. A MACC framework can be the basis of a science-policy process to integrate information about alternative interventions to curb antimicrobial resistance pressure and to identify cost-effective abatement potential in each setting. The framework raises several questions about the technical, economic, behavioural and equity criteria relevant to defining a cap and by extension, global AMU or allowable AMU or budget. In this regard, it is possible to learn from thinking in climate policy to work from what is known as a way to develop consensus on clear evidence gaps and research priorities. A lack of scientific agreement on many elements of AMR is not a reason for inaction.^7^
